# Continuous sensorimotor rhythm based brain computer interface learning in a large population

**DOI:** 10.1038/s41597-021-00883-1

**Published:** 2021-04-01

**Authors:** James R. Stieger, Stephen A. Engel, Bin He

**Affiliations:** 1 Carnegie Mellon University, Pittsburgh, PA USA; 2 University of Minnesota, Minneapolis, MN USA

**Keywords:** Brain-machine interface, Cognitive neuroscience

## Abstract

Brain computer interfaces (BCIs) are valuable tools that expand the nature of communication through bypassing traditional neuromuscular pathways. The non-invasive, intuitive, and continuous nature of sensorimotor rhythm (SMR) based BCIs enables individuals to control computers, robotic arms, wheel-chairs, and even drones by decoding motor imagination from electroencephalography (EEG). Large and uniform datasets are needed to design, evaluate, and improve the BCI algorithms. In this work, we release a large and longitudinal dataset collected during a study that examined how individuals learn to control SMR-BCIs. The dataset contains over 600 hours of EEG recordings collected during online and continuous BCI control from 62 healthy adults, (mostly) right hand dominant participants, across (up to) 11 training sessions per participant. The data record consists of 598 recording sessions, and over 250,000 trials of 4 different motor-imagery-based BCI tasks. The current dataset presents one of the largest and most complex SMR-BCI datasets publicly available to date and should be useful for the development of improved algorithms for BCI control.

## Abstract (summary)

**Table:** 

Measurement(s)	brain measurement
Technology Type(s)	electroencephalography (EEG)
Factor Type(s)	task • mindfulness • brain computer interface • alpha waves
Sample Characteristic - Organism	Homo sapiens

Machine-accessible metadata file describing the reported data: 10.6084/m9.figshare.14153504

## Background & Summary

Millions of individuals live with paralysis^1^. The emerging field of neural prosthetics seeks to provide relief to these individuals by forging new pathways of communication and control^2–6^. Invasive techniques directly record neural activity from the cortex and translate neural spiking into actionable commands such as moving a robotic arm or even individuals’ own muscles^7–13^. However, one major limitation of this approach is that roughly half of all implantations fail within the first year^14^.

A promising alternative to invasive techniques uses the electroencephalogram, or EEG, to provide brain recordings. One popular approach, the sensorimotor rhythm (SMR) based brain computer interface (BCI) detects characteristic changes in the SMR in response to motor imagery^3–6,15,16^. The mu rhythm, one of the most prominent SMRs, is an oscillation in the alpha band and reduces in strength when we move (or think about moving), which is called event-related desynchronization (ERD)^17,18^. The reliable detection of ERD, and its converse event-related synchronization (ERS), enables the intuitive and continuous control of a BCI^19,20^, which has been used to control computer cursors, wheelchairs, drones, and robotic arms^21–28^.

However, mastery of SMR-BCIs requires extensive training, and even after training, 15–20% of the population remain unable to control these devices^27,29^. While the reasons for this remain obscure, the strength of the resting SMR has been found to predict BCI proficiency, and evidence suggests that the resting SMR can be enhanced through behavioral interventions such as mindfulness training^30–32^. Alternatively, better decoding strategies can also improve BCI performance^33,34^.

One encouraging trend in BCI is to use artificial neural networks to decode brain states^13,35–37^. Critically, progress in creating robust and generalizable BCI decoding systems is currently hindered by the limited data available to train these decoding models. Most deep learning BCI studies perform training and testing on the BCI Competition IV datasets^38–42^. While these datasets enable benchmarking performance, the BCI Competition IV datasets 2a and 2b are small (9 subjects, 2–5 sessions) and simple (2a—4 class, no online feedback, 2b—2 class with online feedback, but only 3 motor electrodes)^43^. Most other datasets available online (e.g., those that can be found at http://www.brainsignals.de, or http://bnci-horizon-2020.eu) share similar limitations. Fortunately, two recently published datasets have attempted to address these concerns^44,45^. Cho *et al*. provide a large EEG BCI dataset with full scalp coverage (64 electrodes) and 52 participants, but only 36 minutes and 240 samples of 2-class motor imagery (i.e., left/right hand) per subject. Kaya *et al*. present a larger dataset (~60 hours of EEG recordings from 13 participants over 6 sessions, ~4.8 hours and 4600 trials per participant) with a more complicated design consisting of simple 2-classs motor imagery (left/right hand), 6-class motor imagery (left/right hand, left/right leg, tongue, rest), and motor imagery of individual fingers. However, the motor imaginations were only performed once, which is not suitable for continuous control, and scalp coverage is limited (19 electrodes). Further, neither study provided continuous online feedback.

We recently collected, to our knowledge, the largest SMR-BCI dataset to date^30^. In total, this dataset comprises 62 participants, 598 individual BCI sessions, and over 600 hours of high-density EEG recordings (64 channels) from 269,099 trials. Each individual participant completed 7–11 online BCI training sessions, and the dataset includes on average 4,340 trials and 9.9 hours’ worth of EEG data per participant. This dataset contains roughly 4.5 times as many trials and 10 times as much data as the next largest dataset currently available for public use^44^.

We believe this dataset should be of particular value to the field for four reasons: (1) the amount of EEG data is sufficient to train large decoding models, (2) the sample size permits tests of how well decoding models and signal processing techniques will generalize, (3) the BCI decoding tasks are challenging (e.g., up to 4-class continuous 2D control with online feedback), and (4) the longitudinal study design enables tests of how well decoding models and signal processing techniques adapt to session by session changes. This dataset may additionally provide new insights into how individuals control a SMR-BCI, respond to feedback, and learn to modulate their brain rhythms.

## Methods

### Participants and Experimental procedure

The main goals of our original study were to characterize how individuals learn to control SMR-BCIs and to test whether this learning can be improved through behavioral interventions such as mindfulness training^30^. Participants were initially assessed for baseline BCI proficiency and then randomly assigned to an 8-week mindfulness intervention (Mindfulness-based stress reduction), or waitlist control condition where participants waited for the same duration as the MBSR class before starting BCI training, but were offered a comparable MBSR course after completing all experimental requirements^46^. Following the 8-weeks, participants returned to the lab for 6–10 sessions of BCI training.

All experiments were approved by the institutional review boards of the University of Minnesota and Carnegie Mellon University. Informed consents were obtained from all subjects. In total, 144 participants were enrolled in the study and 76 participants completed all experimental requirements. Seventy-two participants were assigned to each intervention by block randomization, with 42 participants completing all sessions in the experimental group (MBSR before BCI training; MBSR subjects) and 34 completing experimentation in the control group. Four subjects were excluded from the analysis due to non-compliance with the task demands and one was excluded due to experimenter error. We were primarily interested in how individuals learn to control BCIs, therefore analysis focused on those that did not demonstrate ceiling performance in the baseline BCI assessment (accuracy above 90% in 1D control). The dataset descriptor presented here describes data collected from 62 participants: 33 MBSR participants (Age = 42+/−15, (F)emale = 26) and 29 controls (Age = 36+/−13, F = 23). In the United States, women are twice as likely to practice meditation compared to men^47,48^. Therefore, the gender imbalance in our study may result from a greater likelihood of women to respond to flyers offering a meditation class in exchange for participating in our study.

For all BCI sessions, participants were seated comfortably in a chair and faced a computer monitor that was placed approximately 65 cm in front of them. After the EEG capping procedure (see data acquisition), the BCI tasks began. Before each task, participants received the appropriate instructions. During the BCI tasks, users attempted to steer a virtual cursor from the center of the screen out to one of four targets. Participants initially received the following instructions: “Imagine your left (right) hand opening and closing to move the cursor left (right). Imagine both hands opening and closing to move the cursor up. Finally, to move the cursor down, voluntarily rest; in other words, clear your mind.” In separate blocks of trials, participants directed the cursor toward a target that required left/right (LR) movement only, up/down (UD) only, and combined 2D movement (2D)^30^. Each experimental block (LR, UD, 2D) consisted of 3 runs, where each run was composed of 25 trials. After the first three blocks, participants were given a short break (5–10 minutes) that required rating comics by preference. The break task was chosen to standardize subject experience over the break interval. Following the break, participants competed the same 3 blocks as before. In total, each session consisted of 2 blocks of each task (6 runs total of LR, UD, and 2D control), which culminated in 450 trials performed each day.

### Data acquisition

Researchers applied a 64-channel EEG cap to each subject according to the international 10–10 system. The distances between nasion, inion, preauricular points and the cap’s Cz electrode were measured using a measuring tape to ensure the correct positioning of the EEG cap to within ±0.25 cm. The impedance at each electrode was monitored and the capping procedure ensured that the electrodes’ impedance (excluding the rare dead electrode; see artifacts below) remained below 5 kΩ. EEG was acquired using SynAmps RT amplifiers and Neuroscan acquisition software (Compumedics Neuroscan, VA). The scalp-recorded EEG signals were digitized at 1000 Hz and filtered between 0.1 to 200 Hz with an additional notch filter at 60 Hz and then stored for offline analysis. EEG electrode locations were recorded using a FASTRAK digitizer (Polhemus, Colchester, Vermont).

### BCI recordings

Each trial is composed of 3 parts: the inter-trial interval, target presentation, and feedback control. Participants initially saw a blank screen (2 s). They were then shown a vertical or horizontal yellow bar (the target) that appeared in one of the cardinal directions at the edge of the screen for 2 s. After the target presentation, a cursor (i.e., a pink ball) appeared in the center of the screen. The participants were then given up to 6 s to contact the target by moving the cursor in the correct direction by modulating their SMRs. Trials were classified as “hits” when the cursor contacted the correct target and “misses” when the cursor happened to contact one of the other 3 edges of the screen. “Timeouts” occurred when 6 s elapsed without selecting any target. An inter-trial interval of 2 s followed the end of a trial, and then a new trial began. Performance was quantified by a percent valid correct (PVC) metric and was defined as hits/(hits + misses). PVC was averaged across runs for each BCI session. Participants were considered proficient in a given task if their average block or session PVC crossed a given threshold (70% for 1D tasks [LR,UD] and 40% for the 2D task)^49^.

BCI experiments were conducted in BCI2000^20^. Online control of the cursor proceeded in a series of steps. The first step, feature extraction, consisted of spatial filtering and spectrum estimation. During spatial filtering, the average signal of the 4 electrodes surrounding the hand knob of the motor cortex was subtracted from electrodes C3 and C4 to reduce the spatial noise. Following spatial filtering, the power spectrum was estimated by fitting an autoregressive model of order 16 to the most recent 160 ms of data using the maximum entropy method. The goal of this method is to find the coefficients of a linear all-pole filter that, when applied to white noise, reproduces the data’s spectrum. The main advantage of this method is that it produces high frequency resolution estimates for short segments of data. The parameters are found by minimizing (through least squares) the forward and backward prediction errors on the input data subject to the constraint that the filter used for estimation shares the same autocorrelation sequence as the input data. Thus, the estimated power spectrum directly corresponds to this filter’s transfer function divided by the signal’s total power. Numerical integration was then used to find the power within a 3 Hz bin centered within the alpha rhythm (12 Hz).

The translation algorithm, the next step in the pipeline, then translated the user’s alpha power into cursor movement. Horizontal motion was controlled by lateralized alpha power (C4 - C3) and vertical motion was controlled by up and down regulating total alpha power (C4 + C3). These control signals were normalized to zero mean and unit variance across time by subtracting the signals’ mean and dividing by its standard deviation. A balanced estimate of the mean and standard deviation of the horizontal and vertical control signals was calculated by estimating these values across time from data derived from 30 s buffers of individual trial type (e.g., the normalized control signal should be positive for right trials and negative for left trials, but the average of left and right trials should be zero). Finally, the normalized control signals were used to update the position of the cursor every 40 ms.

## Data Records

### Distribution for use

The data files for the large electroencephalographic motor imagery dataset for EEG BCI have been uploaded to the figshare repository^50^. These files can be accessed at 10.6084/m9.figshare.13123148.

### EEG data organization for BCI tasks

This dataset (Citation 51) consists of 598 data files, each of which contains the complete data record of one BCI training session. Each file holds approximately 60 minutes of EEG data recorded during the 3 BCI tasks mentioned above and comprises 450 trials of online BCI control. Each datafile is from one subject and one session and is identified by their subject number and BCI session number.

All data files are shared in .mat format and contain MATLAB-readable records of the raw EEG data and the recording session’s metadata described below. The data in each file are represented as an instance of a Matlab structure named “BCI” having the following key fields “data,” “time,” “positionx,” “positiony,” “SRATE,” “TrialData,” “metadata,” and “chaninfo” (detailed in Table 1). The file naming system is designed to facilitate batch processing and scripted data analysis. The file names are initially grouped by subject number and then session number and can be easily accessed through nested for loops. For example, SX_Session_Y.mat is the filename for subject X’s data record of their Yth BCI training session. Participants are numbered 1 through 62 and sessions are numbered 1 (i.e., the baseline BCI session that occurs 8 weeks prior to the main BCI training) through 11 (or 7 if the participant only completed 6 post intervention training sessions).

**Table 1:** Structure of the data record variable “BCI”.

Subfield Name	Primary Data	Secondary Data	High-Level Description
data	1 × 450 cell	nChannels × nTime matrix	EEG data from each trial of the session
time	1 × 450 cell	1 × nTime vector	vector of the trial time (in ms) relative to target presentation
positionx	1 × 450 cell	1 × nTime vector	X position of cursor during feedback
positiony	1 × 450 cell	1 × nTime vector	Y position of cursor during feedback
SRATE	1 × 1 scalar	N/A	Sampling rate of EEG recording
TrialData	450 × 15 struct	See Table 2	Data structure describing trial level metrics
metadata	12 element struct	See Table 3	Participant and session level demographic information
chaninfo	6 element struct	See Table 4	Information about individual EEG channels

The fields of the structure “BCI” comprising the data record of each file are as follows. The main field is “data,” where the EEG traces of each trial of the session are stored. Since each trial varies in length, the “time” data field contains a vector of the trial time (in ms) relative to target presentation. The two position fields, “positionx” and “positiony,” contain a vector documenting the cursor position on the screen for each trial. “SRATE” is a constant representing the sampling rate of the EEG recording, which was always 1000 Hz in these experiments. “TrialData” is a structure containing all the relevant descriptive information about a given trial (detailed in Table 2). The field “metadata” contains participant specific demographic information (detailed in Table 3). Finally, “chaninfo” is a data structure containing relevant information about individual EEG channels (detailed in Table 4).

**Table 2:** Structure of “BCI” subfield “TrialData”.

Subfield Name	High-Level Description	Values
tasknumber	Identification number for task type	1 = ‘LR’ 2 = ‘UD’ 3 = ‘2D’
runnumber	The run to which a trial belongs	1–18
trialnumber	The trial number of a given session	1–450
targetnumber	Identification number for target presented	1 = right 2 = left 3 = up 4 = down
triallength	The length of the feedback control period in s	0.04 s–6.04 s
targethitnumber	Identification number for target selected by BCI control	1 = right 2 = left 3 = up 4 = down NaN = no target selected; timeout
resultind	Time index for the end of the feedback control portion of the trial	Length(trial) − 1000
result	Outcome of trial: success or failure?	1 = correct target selected 0 = incorrect target selected NaN = no target selected; timeout
forcedresult	Outcome of trial with forced target selection for timeout trials: success or failure?	1 = correct target selected or cursor closest to correct target 0 = incorrect target selected or cursor closest to incorrect target
artifact	Does the trial contain an artifact?	1 = trial contains an artifact identified by technical validation 0 = trial does not contain an artifact identified by technical validation

**Table 3:** Structure of “BCI” subfield “metadata”.

Subfield Name	High-Level Description	Values
MBSRsubject	Did the participant attend the MBSR intervention?	1 = yes 0 = no
meditationpractice	Hours of at-home meditation practiced outside of the MBSR intervention	Hours = MBSR group NaN = control group
handedness	The handedness of the participant	‘L’ = left handed ‘R’ = right handed NaN = data not available
instrument	Did the participant play a musical instrument?	‘Y’ = yes ‘N’ = no ‘U’ = used to NaN = data not available
athlete	Did the participant consider themselves to be an athlete?	‘Y’ = yes ‘N’ = no ‘U’ = used to NaN = data not available
handsport	Did the participant play a hand based sport?	‘Y’ = yes ‘N’ = no ‘U’ = used to NaN = data not available
hobby	Did the participant have a hobby that required fine motor movements of the hands?	‘Y’ = yes ‘N’ = no ‘U’ = used to NaN = data not available
gender	Gender of the participant	‘M’ = male ‘F’ = female NaN = data not available
age	Age of the participant in years	18–63
date	Date of BCI training session	yearmonthday
day	Day of the week of the BCI training session	1–7 starting with 1 = Monday
time	Hour of the start of the BCI training session	7–19

**Table 4:** Structure of “BCI” subfield “chaninfo”.

Subfield Name	Data type	High-level Description
positionsrecorded	bool	1 = subject specific electrode positions were recorded 0 = subject specific electrode positions were not recorded
labels	1 × 62 cell	Electrode names of the EEG channels that form rows of the trial data matrix
noisechan	nNoisyChannels × 1 vector	A vector of the channels labeled as noisy from automatic artifact detection. Empty if no channels were identified as noisy.
electrodes	nChannels × 4 struct (Columns: Label, X, Y, Z)	3D positions of electrodes recorded individually for each session. If this data is not available, generic positions from the 10–10 system will be included.
fiducials	3 × 4 struct (Columns: Label, X, Y, Z)	If positionsrecorded = 1 provides the location of the nasion and left/right preauricular points, otherwise empty
shape	nPoints × 3 struct (Columns: X, Y, Z)	If positionsrecorded = 1 provides the location of the face shape information, otherwise empty.

The “data” field contains the recording session’s EEG data in the format of a 1 × nTrials cell array, where each entry in the cell array is a 2D Matlab array of size nChannels × nTime. The number of trials per session, nTrials, is nearly always 450. Each row of a trial’s 2D data matrix is the time-series of voltage measurements (in μV) from a single EEG input lead such as C3 or C4. The “time” field contains the time data in the format of a 1 × nTrials cell array, where each entry is a 1 × nTime vector of the time index of the trial referenced to target presentation. Each trial begins with a 2 s inter-trial interval (index 1, t = −2000ms), followed by target presentation for 2 s (index 2001, t = 0). Feedback begins after target presentation (index 4001, t = 2000ms) and continues until the end of the trial. The end of the trial can be found in the “resultind” subfield of “TrialData” (see below). A post-trial interval of 1 s (1000 samples) follows target selection or the timeout signal.

While the length of the trial may vary, nTime (the number of time samples of the trial), the number of channels, nChannels, is always 62. The ordering of the channels can be found in the “chaninfo” structure’s subfield “label”, which lists the channel names of the 62 electrodes. If individual electrode positions were recorded, the subfield “positionsrecorded” will be set to 1 and the “electrodes,” “fiducials,” and “shape” subfields will be populated according to this information. “Electrodes” contains the electrode labels and their X, Y, and Z coordinates. The information in the “fiducials” subfield contains the X, Y, and Z locations of the nasion as well as the left and right preauricular points. The shape of the face was recorded by sweeping from ear to ear under the chin and then drawing arcs horizontally across the brow and vertically from the forehead to chin. This information is contained in the “shape” subfield. If the session specific individual electrode positions are not available, “positionsrecorded” will be set to zero, the “fiducials” and “shape” subfields will be empty, and the “electrodes” subfield will include generic electrode locations for the 10–10 system positioning of the Neuroscan Quik-Cap. Finally, the subfield “noisechan” identifies which channels were found to be particularly noisy during the BCI session (see Technical Validation) and can be used for easy exclusion of this data or channel interpolation.

The position subfields of the “BCI” structure, “positionx” and “positiony,” provide the cursor positions throughout the trial in the format of a 1 × nTrials cell array. Each entry of this cell array is a 1 × nTime vector of the horizontal or vertical position of the cursor throughout the trial. Positions are provided by BCI2000 starting with feedback control (index 4001) and continue through the end of the trial (TrialData(trial).resultind). The inter-trial intervals before and after feedback are padded with NaNs. The positional information ranges from 0 (left/bottom edge of the screen) to 1 (right/top edge of the screen), with the center of the screen at 0.5. During 1D tasks (LR/UD), the orthogonal position remains constant (e.g., in the LR task, positiony = 0.5 throughout the trial).

The “TrialData” structure subfield of “BCI” provides valuable information for analysis and supervised machine learning (detailed in Table 2). This structure contains the subfields “tasknumber,” “runnumber,” “trialnumber,” “targetnumber,” “triallength,” “targethitnumber,” “resultind,” “result,” “forcedresult,” and “artifact.” Tasknumber is used to identify the individual BCI tasks (1 = ‘LR’, 2 = ‘UD’, 3 = ‘2D’). Targetnumber is used to identify which target was presented to the participants (1 = right, 2 = left, 3 = up, 4 = down). “Triallength” is the length of the feedback control period in seconds, with the maximum length of 6.04 s occurring in timeout trials. “Resultind” is the time index that indicates when the feedback ended (when a target was selected or the trial was considered a timeout). To easily extract the feedback portion of the EEG data, you simply define an iterator variable (e.g., trial = 5) and index into the data subfield [e.g., trial_feedback = BCI.data{trial}(4001:BCI.TrialData(trial).resultind)]. “Targethitnumber” identifies which target was selected during feedback and takes the same values as “targetnumber” mentioned above. These values will be identical to “targetnumber” when the trial is a hit, different when the trial is a miss, and NaN when a trial is a timeout. “Result” is a label for the outcome of the trial which takes values of 1 when the correct target was selected, 0 when an incorrect target is selected and NaN if the trial was labeled as a timeout. “Forcedresult” takes the same values as before, however in timeout trials, the final cursor position is used to select the target closest to the cursor thereby imposing the BCI system’s best guess onto timeout trials. Finally the “artifact” subfield of the “TrialData” structure is a bool indicating whether an artifact was present in the EEG recording (see Technical Validation).

Lastly, the “metadata” field of the “BCI” data structure includes participant and session level demographic information (detailed in Table 3). “Metadata” contains information related to the participant as an individual, factors that could influence motor imagery, information regarding the mindfulness intervention, and individual session factors. Participant demographic information includes “age” (in years), “gender” (‘M’, ‘F’ NaN if not available), and “handedness” (‘R’, ‘L’, NaN). Motor imagery factors include “instrument”, “athlete”, “handsport”, and “hobby.” These factors are reported as ‘Y’ for yes, ‘N’ for no, ‘U’ for used to, and NaN if not available. Subfields for the mindfulness intervention include “MBSRsubject” “meditationpractice”. Finally, session factors included “date, “day”, and “time”.

While the data is formatted for ease of use with Matlab, this software is not required. The data structure is compatible with Octave. However, to open the data in python, the fields of the BCI structure should be saved in separate variables. For example, the cell BCI.data can be saved as ‘data’ in a.mat file and then loaded into python as an ndarray with the scypy.io function loadmat. The TrialData structure can also be converted to a matrix and cell of column names then converted to a pandas dataframe.

## Technical Validation

**Fig. 1:**
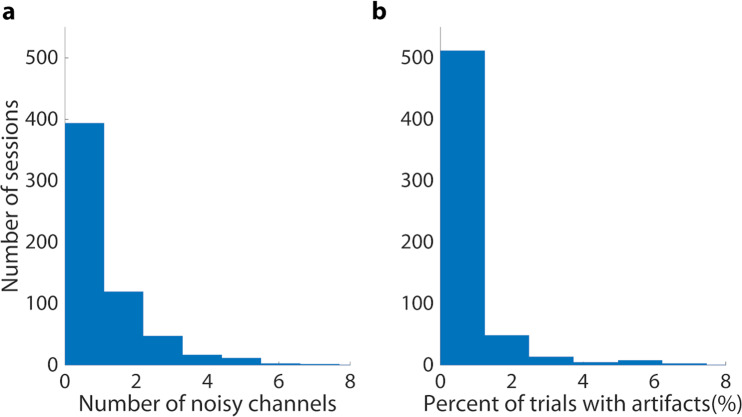
Histograms of data records containing noisy data (**a**) Most data records have few channels automatically labeled as containing excessive variance. (**b**) Most data records have few trials containing artifacts.

Automatic artifact detection was used to demonstrate the high quality of the individual data records^45,51^. First, the EEG data were bandpass filtered between 8 Hz and 30 Hz. If the variance (across time) of the bandpass filtered signals exceeded a z-score threshold of 5 (z-scored between electrodes), these electrodes were labeled as artifact channels for a given session. This information can be found in BCI.chaninfo.noisechan. Overall, only 3% of sessions contained more than 4 artifact channels (Fig. 1a). These channels were excluded from the subsequent artifact detection step and their values were interpolated spatially with splines for the ERD topographies shown below^52^. Then, if the bandpass filtered data from any remaining electrode crossed a threshold of ±100 μV throughout a trial, this trial was labeled as containing an artifact. Artifact trials are labeled in the TrialData structure (BCI.TrialData.artifact). Again, only 3% of sessions had more than 5% of trials labeled as containing artifacts (Fig. 1b).

**Fig. 2:**
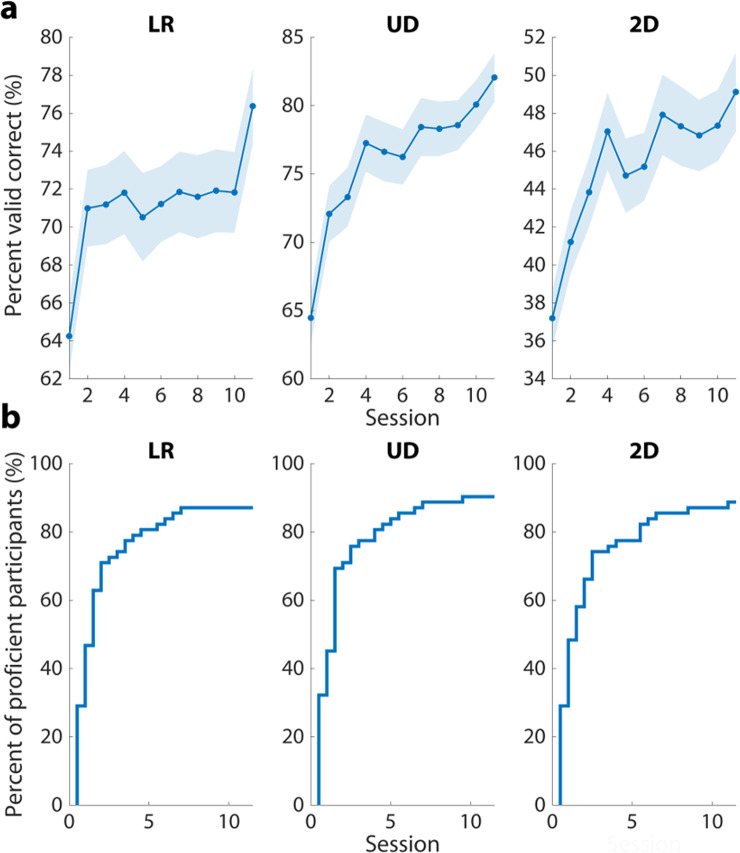
Online BCI performance across sessions (**a**) Following the baseline assessment, the average online BCI performance exceeds chance level across all tasks suggesting decodable motor imagery patterns are present in the EEG data records. As BCI training continues, participants produce better linearly classifiable signals in the alpha band. The shaded area represents ±1 standard error of the mean (SEM). (**b**) Group-level BCI proficiency throughout training. This curve shows the percentage of participants that are proficient in BCI control as a function of BCI training session. At the end of training, approximately 90% of all subjects were considered proficient in each task of BCI control.

More research is needed to understand how individuals learn to control brain computer interfaces^27^, and this dataset provides a unique opportunity to study this process. Not only does the online BCI performance demonstrate that motor imagery can be decoded from the EEG data records, it also shows that the decidability of the participants’ EEG rhythms improves as individuals learn to control the device (Fig. 2a). BCI proficiency is also an active area of study^53^, and, by the end of training, roughly 90% of all the participants in our study were considered proficient in each task (Fig. 2b). However, the sample size of this dataset is large enough that 7–8 participants can still be examined for BCI inefficiency in each task. This dataset additionally enables the study of the transition from BCI inefficiency to proficiency.

**Fig. 3:**
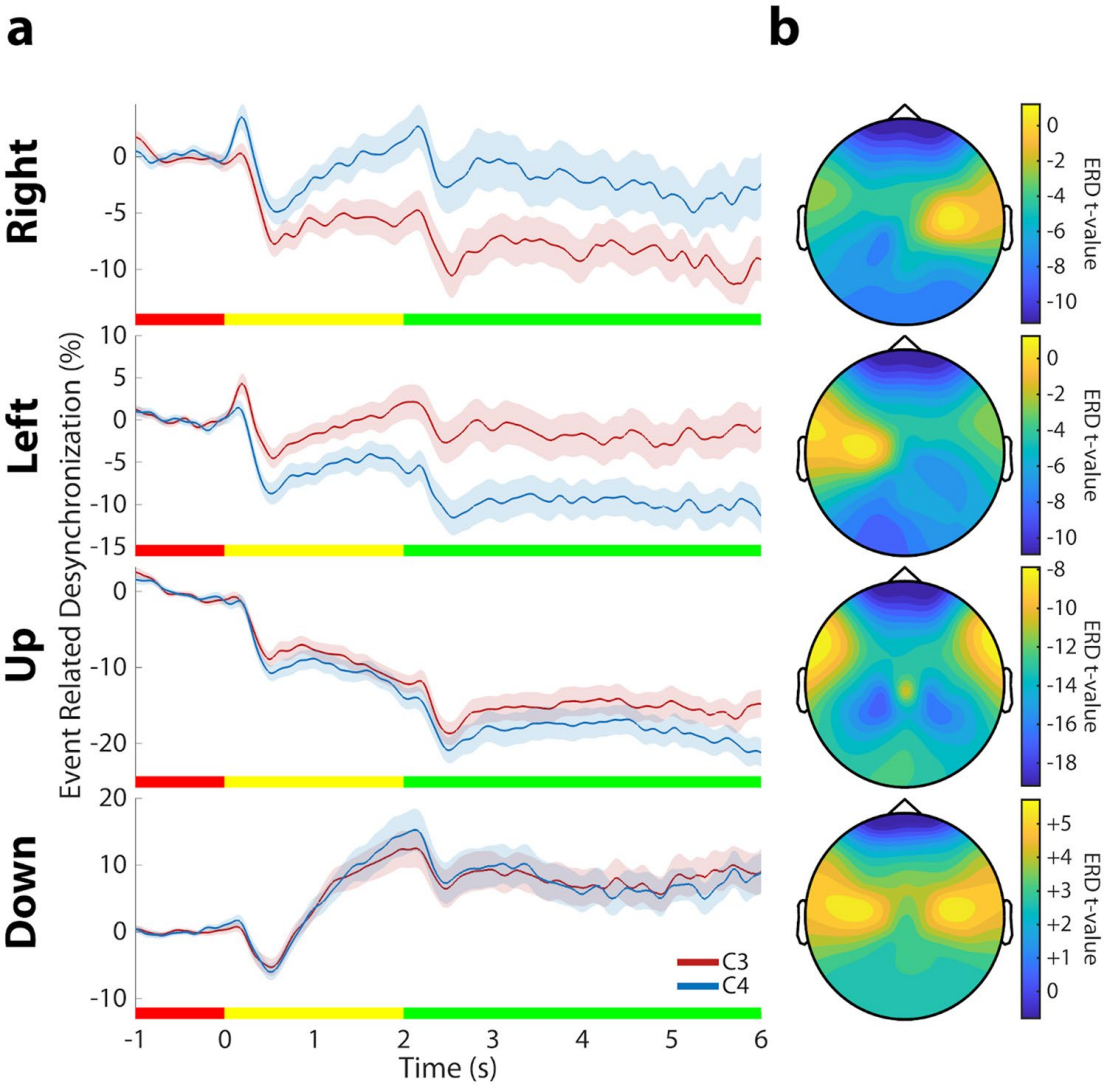
Event-related desynchronization during BCI control (**a**) ERD averaged across participants and training sessions. These curves show the change in alpha power (8–14 Hz) over the left (C3; red) and right (C4; blue) motor cortex relative to the power in the inter-trial interval. The ERD curves show the expected responses to motor imagery such as stronger desynchronization (negative values) in the contralateral motor cortex during motor imagery and stronger synchronization during rest (positive values). The rectangles at the bottom of each plot display the different phases of each trial with red, yellow, and green representing the inter-trial interval, target presentation, and feedback control periods, respectively. The shaded are represents ± 1 SEM. (**b**) Topographies of ERD values across the cortex for each trial type. T-values were found by testing the ERD values across the population in our study. Cooler colors represent more desynchronization during motor imagery and warmer colors represent more synchronization.

Event-Related Desynchronization (ERD) was calculated to verify the quality of the EEG signals collected during BCI control^17,45^. To calculate ERD for each channel, the EEG data were first highpass filtered above 1 Hz to remove drifts, then bandpass filtered between 8 Hz and 14 Hz^52^. The Hilbert transform was then applied to all of the trials and the absolute magnitude was taken for each complex value of each trial. The magnitudes of the Hilbert-transformed samples were averaged across all trials. Finally, these averages were baseline corrected to obtain a percentage value for ERD/ERS according to the formula ${\rm{ERD}} \% =\frac{A-R}{R}\ast 100 \%$, where A is each time sample and R is the mean value of the baseline period (−1 s to 0 s). The participant averaged ERD signal for channels C3 and C4, and for each trial type, are shown in Fig. 3a. ERD values were averaged over the feedback portion of trials to create the topography images in Fig. 3b. The ERD curves and scalp topographies show the expected responses to motor imagery such as stronger desynchronization in the contralateral motor cortex during motor imagery and stronger synchronization during rest.

## Data Availability

The code used to produce the figures in this manuscript is available at https://github.com/bfinl/BCI_Data_Paper.
